# Clinical, Serological, and Histopathological Similarities Between Severe COVID-19 and Acute Exacerbation of Connective Tissue Disease-Associated Interstitial Lung Disease (CTD-ILD)

**DOI:** 10.3389/fimmu.2020.587517

**Published:** 2020-10-02

**Authors:** Daniel Gagiannis, Julie Steinestel, Carsten Hackenbroch, Benno Schreiner, Michael Hannemann, Wilhelm Bloch, Vincent G. Umathum, Niklas Gebauer, Conn Rother, Marcel Stahl, Hanno M. Witte, Konrad Steinestel

**Affiliations:** ^1^ Department of Pulmonology, Bundeswehrkrankenhaus Ulm, Ulm, Germany; ^2^ Clinic of Urology, University Hospital Augsburg, Augsburg, Germany; ^3^ Department of Radiology, Bundeswehrkrankenhaus Ulm, Ulm, Germany; ^4^ Department of Laboratory Medicine, Bundeswehrkrankenhaus Ulm, Ulm, Germany; ^5^ Department of Molecular and Cellular Sport Medicine, German Sport University Cologne, Cologne, Germany; ^6^ Institute of Pathology and Molecular Pathology/Study Center of the German Registry of COVID-19 Autopsies (DeRegCOVID), Bundeswehrkrankenhaus Ulm, Ulm, Germany; ^7^ Department of Hematology and Oncology, University Hospital Schleswig-Holstein Campus Luebeck, Luebeck, Germany; ^8^ Department of Hematology and Oncology, Bundeswehrkrankenhaus Ulm, Ulm, Germany

**Keywords:** autoimmunity, connective tissue disease, SARS-CoV-2, coronavirus disease 2019, autoantibodies

## Abstract

**Background and Objectives:**

Understanding the pathophysiology of respiratory failure in coronavirus disease 2019 (COVID-19) is indispensable for development of therapeutic strategies. Since we observed similarities between COVID-19 and interstitial lung disease in connective tissue disease (CTD-ILD), we investigated features of autoimmunity in SARS-CoV-2-associated respiratory failure.

**Methods:**

We prospectively enrolled 22 patients with RT-PCR-confirmed SARS-CoV-2 infection and 10 patients with non-COVID-19-associated pneumonia. Full laboratory testing was performed including autoantibody (AAB; ANA/ENA) screening using indirect immunofluorescence and immunoblot. Fifteen COVID-19 patients underwent high-resolution computed tomography. Transbronchial biopsies/autopsy tissue samples for histopathology and ultrastructural analyses were obtained from 4/3 cases, respectively.

**Results:**

Thirteen (59.1%) patients developed acute respiratory distress syndrome (ARDS), and five patients (22.7%) died from the disease. ANA titers ≥1:320 and/or positive ENA immunoblots were detected in 11/13 (84.6%) COVID-19 patients with ARDS, in 1/9 (11.1%) COVID-19 patients without ARDS (p = 0.002) and in 4/10 (40%) patients with non-COVID-19-associated pneumonias (p = 0.039). Detection of AABs was significantly associated with a need for intensive care treatment (83.3 *vs.* 10%; p = 0.002) and occurrence of severe complications (75 *vs.* 20%, p = 0.03). Radiological and histopathological findings were highly heterogeneous including patterns reminiscent of exacerbating CTD-ILD, while ultrastructural analyses revealed interstitial thickening, fibroblast activation, and deposition of collagen fibrils.

**Conclusions:**

We are the first to report overlapping clinical, serological, and imaging features between severe COVID-19 and acute exacerbation of CTD-ILD. Our findings indicate that autoimmune mechanisms determine both clinical course and long-term sequelae after SARS-CoV-2 infection, and the presence of autoantibodies might predict adverse clinical course in COVID-19 patients.

## Introduction

Coronavirus disease 2019 (COVID-19), caused by severe acute respiratory syndrome coronavirus 2 (SARS-CoV-2), has caused or contributed to hundreds of thousands of deaths and led to almost complete shutdown of social and economic life in many countries (1). Based on what is currently known about epidemiology, COVID-19 is associated with a mortality rate between 1 and 7% (2). Major cause of death in COVID-19 infections is acute respiratory failure (acute respiratory distress syndrome, ARDS), but the exact mechanism of how COVID-19 leads to ARDS is unclear. In most reported morphological analyses, the authors describe diffuse alveolar damage (DAD) with an early edematous phase followed by hyaline membrane formation, desquamation of pneumocytes, and an increased interstitial mononuclear infiltrate in severe SARS-CoV-2 infection (3). In one case, Tian et al. report loose intra-alveolar fibromyxoid proliferation reminiscent of organizing pneumonia (OP) (4). Such combined histological patterns of (organizing) DAD and OP, summarized by some authors under the term acute fibrinous organizing pneumonia (AFOP), have also been observed in interstitial lung disease associated with systemic lupus erythematosus (SLE), dermatomyositis, and progressive systemic sclerosis (PSS) (5–7). This is of special relevance since both organizing DAD as well as CTD-ILD may evolve to pulmonary fibrosis, and long-term effects of COVID-19 are so far unknown. Only recently, upregulation of fibrosis-associated gene expression in COVID-19 has been described (8).

Most CTDs are defined by the presence of specific antinuclear autoantibodies (ANAs), several of which have been identified and summarized under the historic term extractable nuclear antibodies (ENAs), such as anticentromer antibodies (CENP-B), PM-Scl, SS-B/La, Jo-1, and Scl-70 (9). Only recently, the presence of such autoantibodies has been described in cases of severe COVID-19, but the exact relevance of this finding remains unclear (10, 11).

Taken together, since available data suggests histomorphological as well as pathophysiological similarities between COVID-19-associated ARDS and lung manifestations of autoimmune disease, we hypothesized that a dysregulated immune response upon SARS-CoV-2 infection might show similarities to acute exacerbation of CTD-ILD which might shed some light on the mechanism of lung damage in COVID-19.

## Methods

In this prospective trial we consecutively included all patients with positive SARS-CoV-2-RT-PCR (mucosal swab, pharyngeal or bronchoalveolar lavage) admitted to the Bundeswehrkrankenhaus (Armed Forces Hospital) Ulm in March and April 2020 after obtaining informed consent. Suspected cases without RT-PCR-confirmed SARS-CoV-2 infection were excluded from the study. A group of 10 patients with non-COVID-19-associated pneumoniae served as control group for serological analyses (**Supplementary Table 1**). Patients or their relatives had given written informed consent to routine diagnostic procedures (serology, bronchoscopy, radiology) as well as (partial) autopsy in the case of death, respectively, as well as to the scientific use of data and tissue samples in the present study. This project was approved by the local ethics committee of the University of Ulm (ref. no. 129-20) and conducted in accordance with the Declaration of Helsinki.

### Clinical Characteristics

We collected clinical information from electronic patient files. Data included disease-related events, preexisting comorbidities, imaging, treatment approaches (**Supplementary Table 2**), and clinical follow-up. The “Berlin definition’’ was used to categorize ARDS (12). The Horovitz quotient (PaO_2_/FiO_2_) was assessed in all ARDS cases based on arterial blood gas analysis.

### ICU Treatment

During ICU treatment, ventilation parameters, duration of invasive ventilation, catecholamine support, prone positioning, Murray lung injury score and the need of additional temporary dialysis were continuously assessed (**Supplementary Table 3**) (13). A profitable trial of prone positioning was defined by an increasing Horovitz quotient of 30 mmHg or more. One entire trial covered 16 h of sustained prone positioning.

### Serology/Laboratory Values

Blood samples for serology and monitoring of laboratory values were taken at hospital admission and during ward/ICU treatment, respectively. Laboratory values included possible predictors of outcome in COVID-19 patients such as lymphocyte count, fibrinogen, D-dimers, ferritin, lactate dehydrogenase (LDH), and bilirubin. We also assessed troponin-T levels as a marker for cardiac events and infection-associated parameters (neutrophils, interleukin-6 (IL-6), C-reactive protein (CRP), and procalcitonin (PCT)). Cut-off values, median, and range for these parameters are summarized in **Supplementary Table 4**.

### ANA/ANCA/ENA Testing

Initial screenings for ANA and ANCA (p-ANCA, c-ANCA, x-ANCA, Anti-PR3, Anti-MPO) were performed by IIF using patient sera on Hep-2 cells and primate liver tissue slides (ANA) as well as ethanol- and formol-fixed granulocytes and purified PR-3 and MPO antigens (ANCA) on glass slides (Euroimmun AG, Lübeck, Germany) according to the manufacturer’s protocols (14, 15). In all cases, presence of specific anti-ENA autoantibodies (anti-Sm, anti-SS-A/Ro, anti-SS-B/La, anti-Scl-70, anti-centromere, anti-Jo1, anti-Mi-2, anti-U1-RNP, anti-Ro-52, anti-PM-Scl, anti-CNP B, anti-PCNA, anti-dsDNA, anti-nucleosome, anti-histone, anti-ribosomal P-protein, anti-AMA-M2) was assessed by semiquantitative immunoblot (Anti-ENA Profile 3; Euroimmun AG, Lübeck, Germany) irrespective of the initial screening result. Following previously published guidelines, ANA titers ≥1:320 with or without positive ENA immunoblot **or** ANA titers of 1:100 with positive ENA immunoblot were regarded as positive (9, 16). Laboratory testing was performed by investigators who were blinded to patient status, and in cases with unclear/borderline results in either ANA screening or ENA subtyping, tests were repeated on a new sample, and results were verified by an external reference laboratory. Quality and reliability of ANA/ANCA/ENA testing in our institution have been evaluated through regular interlaboratory ring trials.

### Imaging

Imaging was performed on a Somatom Force Scanner (Dual Source Scanner 2*192 slices, Siemens, Erlangen, Germany) in accordance to the guidelines of the German Radiological Society and our hospital’s COVID-19 guidelines, using low-dose CT (computed tomography) with high-pitch technology (17). The following parameters were used: Tube voltage: 100 kV with tin filtering, tube current: 96 mAs with tube current modulation. In two cases examination was performed as a non-contrast enhanced full-dose protocol because of suspected ILD, in one case as a contrast-enhanced CT scan to exclude pulmonary thromboembolism. X-ray examinations were performed at the respective wards as bed-side X-ray examinations (Mobilett Mira Max, Siemens, Erlangen, Germany) as a single anterior–posterior view. The CT images were evaluated according to the Expert Consensus Statement of the RSNA and classified as typical, indeterminate, atypical, and negative appearance for COVID-19 (17, 18).

### Histology and Immunohistochemistry

Lung tissue specimens were obtained as transbronchial biopsies in four cases. In three deceased patients, partial autopsies were performed, and lung, heart, and liver tissues were sampled extensively. Specimens were stained with hematoxylin–eosin (HE), Phosphotungstic-Acid–Hematoxylin (PTAH), Elastica-van-Gieson (EvG) and Masson-Goldner (MG). Furthermore, immunohistochemistry for CD3, CD68, CK7, CMV, and EBV was performed using prediluted antibodies on a VENTANA benchmark autostainer (Roche Tissue Diagnostics, Mannheim, Germany) following routine protocols.

### Electron Microscopy

Lung, heart, and liver tissues were immersion-fixed with 4% paraformaldehyde in 0.1 M PBS, pH 7.4. After several time washing in 0.1 M PBS, tissue was osmicated with 1% OsO4 in 0.1 M cacodylate and dehydrated in increasing ethanol concentrations. Epon infiltration and flat embedding were performed following standard procedures. Methylene blue was used to stain semithin sections of 0.5 µm. Seventy to ninety-nanometer-thick sections were cut with an Ultracut UCT ultramicrotome (Fa. Reichert) and stained with 1% aqueous uranylic acetate and lead citrate. Samples were studied with a Zeiss EM 109 electron microscope (Fa. Zeiss) coupled to a Megaview III Soft Imaging System camera analySIS^®^ software both from Fa. (Soft Imaging System GmbH).

### Statistical Methods

Descriptive statistical methods were used to summarize the data. Medians and interquartile ranges were used to announce results. Absolute numbers and percentages were employed to represent categorial variables. Student’s t-test was used for the comparison of continuous variables, while Chi-Square-Test/Fisher’s test was used for categorial variables. All statistical analyses were conducted using GraphPad PRISM 6 (GraphPad Software Inc., San Diego, CA, USA). A p-value <0.05 was regarded as statistically significant.

## Results

### Baseline Clinical Characteristics of the Study Cohort

**Figure 1:**
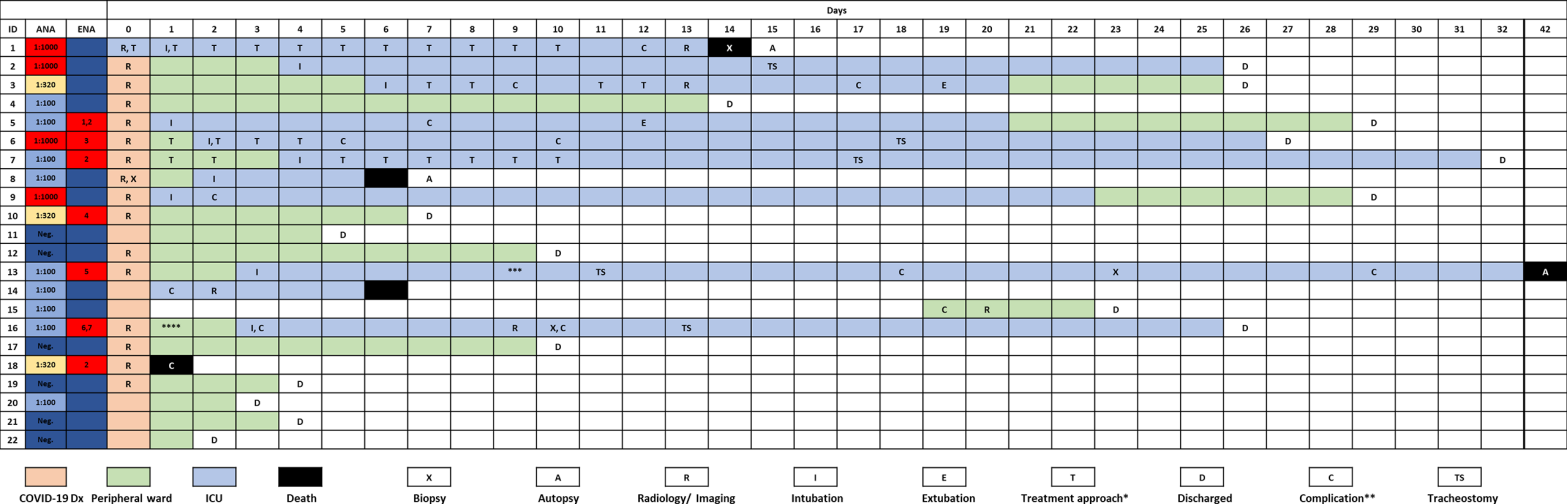
Timeline of the complete study cohort. *, treatment modalities are summarized in **Supplementary Table 1**; **, case-related complications are summarized in **Supplementary Table 5**; ***, patient was transferred to another institution because vv-ECMO was required; ****, patient w/chronic lymphocytic leukemia (CLL) and antibody deficiency syndrome. Rheumatoid factor was positive in this case. ^1–7^Specific ENAs: ^1^anti-SS-B, ^2^anti-PM-Scl, ^3^anti-Jo, ^4^anti-CENP, ^5^anti-Scl-70, ^6^anti-Nucleosome, ^7^anti-dsDNA.

Baseline clinical characteristics of SARS-CoV-2 infected patients are briefly summarized in **Table 1**, and we show a timeline of the complete study cohort with all relevant events in **Figure 1**. Clinical characteristics of non-COVID-19-associated pneumonia patients (controls) are summarized in **Supplementary Table 1**. Median age at initial diagnosis was 69.0 years (range, 28–88 years). The majority of patients were male (12/22 cases; 54.5%). Most frequent preexisting comorbidities were cardiovascular risk factors (13/22, 59.1%) and established cardiovascular disease (10/22, 45.5%). Preexisting rheumatic disease was present in 2/22 cases (9.1%): one patient (#16) had rheumatoid factor-positive rheumatoid arthritis, the other patient (#1) had a history of rheumatic disease and associated treatment which could not be evaluated in more detail. Treatment approaches and drug-related toxicities are summarized in **Supplementary Table 2**. Bacterial superinfection was suspected in 10/22 (45.5%) cases by clinical course, imaging, and laboratory values. Antibiotic treatment approaches are summarized in **Supplementary Table 5**. There was an overall high rate of complications compared to regular (non-COVID) ARDS patients (**Supplementary Table 6** **)**. One patient was temporarily transferred to another hospital because vv-ECMO (veno-venous extracorporeal membrane oxygenation) was required. After a median follow-up period of 64.5 days (range, 1–81 days), five patients (22.7%) had died from the disease.

**Table 1:** Baseline clinical characteristics of all COVID-19 patients and stratified according to development of ARDS.

Characteristics	All patientsN = 22	No ARDSN = 9	ARDSN = 13	p-value
**Age**	**n = 22**	**n = 9**	**n = 13**	
years; median (range)	69 (28–88)	57 (28–88)	71 (53–87)	**0.044**
**Sex**		**n = 9**	**n = 13**	
Female	10 (45.5%)	8 (88.9%)	2 (15.4%)	**0.002**
Male	12 (54.5%)	1 (11.1%)	11 (84.6%)	
**Preexisting diseases**	**n = 22**	**n = 9**	**n = 13**	
0	6 (27.3%)	5 (55.6%)	1 (7.7%)	**0.023**
≥1	16 (72.7%)	4 (44.4%)	12 (92.3%)	
**Type of preexisting disease**	**n = 22**	**n = 9**	**n = 13**	
Cardiovascular risk factors^1^	13 (59.1%)	4 (44.4%)	9 (69.2%)	0.384
Cardiovascular disease^2^	10 (45.5%)	4 (44.4%)	6 (46.2%)	1
Oncological disease	5 (22.7%)	1 (11.1%)	4 (30.8%)	0.36
Rheumatic disease	2 (9.1%)	–	2 (15.4%)	0.494
**Duration of symptomatic disease (after positive SARS-CoV-2 testing)**	**n = 22**	**n = 9**	**n = 13**	
days; median (range)	15 (1–28)	14 (12–22)	17 (1–28)	0.315
**SARS-CoV-2 detection method**	**n = 22**	**n = 9**	**n = 13**	
Mucosal swab	17 (77.3%)	8 (88.9%)	9 (69.2%)	0.36
Pharyngeal lavage	4 (18.2%)	1 (11.1%)	3 (23.1%)	0.616
Bronchoalveolar lavage	1 (4.5%)	–	1 (7.7%)	1
**Imaging at initial diagnosis**	**n = 22**	**n = 9**	**n = 13**	
Chest X-ray	3 (13.6%)	1 (11.1%)	2 (15.4%)	1
CT scan	15 (68.2%)	4 (44.4%)	11 (84.6%)	0.074
No imaging at initial diagnosis	4 (18.2%)	4 (44.4%)	–	**0.017**
**Radiological features at initial diagnosis (CT Scan)^3^ **	**n = 15**	**n = 4**	**n = 11**	
Typical	8 (53.3%)	2 (50.0%)	6 (54.5%)	1
Indeterminate	1 (6.7%)	–	1 (9.1%)	1
Atypical	2 (13.4%)	–	2 (18.2%)	1
Negative for pneumonia	4 (26.7%)	2 (50.0%)	2 (27.3%)	0.517
**ICU treatment**	**n = 22**	**n = 9**	**n = 13**	
Yes	11 (50%)	–	11 (84.6%)	**<0.001**
No	11 (50%)	9 (100.0%)	2 (15.4%)	
**Respiration**	**n = 22**	**n = 9**	**n = 13**	
Breathing spontaneously	9 (40.9%)	9 (100.0%)	–	**<0.001**
Oxygen support	3 (13.6%)	–	3 (23.1%)	
Invasive ventilation	10 (45.5%)	–	10 (76.9%)	
**LDH**	**n = 20**	**n=7**	**n=13**	
U/L; median (range)	297 (167–754)	194 (167–254)	365 (248–754)	**0.002**
**C-reactive protein**	**n = 20**	**n = 7**	**n = 13**	
mg/dl; median (range)	7.3 (0.1–42.1)	0.5 (0.1–25.5)	11.7 (2–42.1)	**0.038**
**IL-6**	**n = 21**	**n = 8**	**n = 13**	
pg/ml; median (range)	61 (2–2205)	12 (2–198)	258 (30–2205)	**0.046**
**ANA/ENA (IIF+IB)**	**n = 22**	**n = 9**	**n = 13**	
ANA/ENA neg.^4^	10 (45.5%)	8 (88.9%)	2 (15.4%)	**0.002**
ANA/ENA pos.^5^	12 (54.5%)	1 (11.1%)	11 (84.6%)	
**Outcome**	**n = 22**	**n = 9**	**n = 13**	
Follow-up: days; median (range)	64.5 (1–81)	72 (47–75)	59 (1–81)	0.069
Dead from disease	5 (22.7%)	–	5 (38.5%)	0.054
Severe complications^6^	10 (45.5%)	1 (11.1%)	9 (69.2%)	**0.012**

ANA, antinuclear autoantibody; ARDS, acute respiratory distress syndrome; CT, computed tomography; ENA, extractable nuclear antigen; IB, immunoblot; IIF, indirect immunofluorescence; IL-6, interleukin 6; LDH, lactate dehydrogenase; na, not applicable; SARS-CoV-2, severe acute respiratory syndrome coronavirus 2.

^1^diabetes mellitus, dyslipidemia, arterial hypertension, obesity, nicotine abuse; ^2^coronary disease, post-myocardial infarction, peripheral arterial vaso-occlusive disease, post-stroke, atherosclerosis; ^3^Radiological features have been classified referring to Simpson et al., Radiology: Cardiothoracic Imaging 2.2 (2020): e200152; ^4^ANA/ENA negative: ANA titer <1:320 and negative ENA immunoblot; ^5^ANA/ENA positive: ANA titer ≥1:320 and/or positive ENA immunoblot; ^6^Severe complications are summarized in **Supplementary Table 5**.

Significant results (p < 0.05) are highlighted in bold.

### ARDS/Non-ARDS Patients

Thirteen of 22 COVID-19 cases (59.1%) and 1/10 patients (10%) with non-COVID-19-associated pneumonia presented with or developed ARDS according to the “Berlin definition’’(12), and intensive care unit (ICU) treatment was required in 11/22 (50%) of COVID-19 patients, and one patient with non-COVID-19-associated pneumonia. COVID-19 patients who developed ARDS were significantly older, and most of them were male (p = 0.044 and p = 0.002, **Table 1**). Furthermore, these patients presented with more preexisting comorbidities (p = 0.023). ARDS was significantly associated with ICU treatment, occurrence of severe complications and (invasive) ventilation in COVID-19 positive patients (p < 0.001, p = 0.012 and p < 0.001, respectively). The Murray lung injury score was calculated for all patients who underwent invasive ventilation and revealed moderate or severe ARDS in 8/10 cases (80.0%) (13). All five COVID-19-associated deaths occurred in the ARDS group.

**Figure 2:**
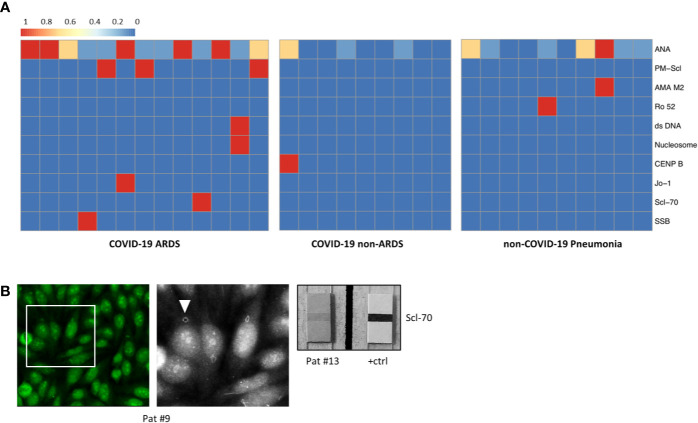
**(A)** Heatmap showing the distribution and subtypes of autoantibodies in the group of COVID-19 patients with ARDS (left), COVID-19 patients without development of ARDS (center), and non-COVID-19 pneumonia patients (right; clinical data of the non-COVID-19 pneumonia controls are summarized in **Supplementary Table 6**). **(B)** IIF image (left) with fine granular/nucleolar staining pattern and clearly visible “rings and rods” (arrowhead in high magnification inset) (patient #9). Right: positive ENA immunoblot for Scl-70 (patient #13).

Laboratory values for LDH, CRP, and IL-6 were significantly higher in the group of COVID-19-positive patients who developed ARDS compared to COVID-19 patients with mild clinical course. However, LDH, CRP, and IL-6 values were not significantly different between COVID-19 ARDS patients and patients with non-COVID-19-associated pneumonia (each p > 0.05, **Table 1** and **Supplementary Table 1**). Initial ANA screening by IIF showed titers ≥1:100 in all COVID-19 ARDS patients (100%) but in 4/9 (44.4%) COVID-19 patients without ARDS. Among the group of non-COVID-19-associated pneumonias, the initial ANA screening by IIF showed titers ≥1:100 in 7/10 patients (70%). ANCA screening was completely negative in all investigated COVID-19 cases but positive in 1/10 patients (10%) with non-COVID-19-associated pneumonia. Specific autoantibodies could be detected by ENA immunoblot in 6/12 COVID-19 ARDS patients (50%) but in 1/9 COVID-19 non-ARDS patients (11.1%) and 2/10 patients (20%) with non-COVID-19-associated pneumonia. One patient from the ARDS group (#9, ANA titer 1:1000) showed borderline positivity for anti-RNA polymerase III (RP155) autoantibodies only after reference laboratory testing and was therefore classified as negative. The distribution and type of ANA/ENA among the different subgroups are shown in **Figure 2A**, while representative images of IIF and IB are shown in **Figure 2B**. PM-Scl was the most commonly detected autoantibody (3/7 cases) and could only be detected in the COVID-19 ARDS group.

Taken together, applying established diagnostic criteria for ANA/ENA screening as described above (16), 11/13 ARDS patients (84.6%) and 1/11 non-ARDS patients (11.1%) were classified as ANA-positive (p = 0.002), while 4/10 (40%) of non-COVID-19-associated pneumonia patients were classified as positive (p = 0.039). Of the two COVID-19 patients with a history of rheumatic disease (#1 and 16), one patient (#1) showed high ANA titer in IIF (1:1000), while the other patient (#16) had detectable anti-Nucleosome/anti-dsDNA antibodies.

### ANA and Clinical Course in COVID-19 Patients

Detection of ANA was associated with higher age and male sex, although not significant. ANA positivity, however, was associated with a necessity of assisted/invasive ventilation, ICU treatment (both p = 0.002) and occurrence of severe complications (p = 0.03). Typical or atypical patterns in CT imaging were not different between patients with and without ANAs. While there were no significant differences in serum levels of CRP and IL-6, LDH levels were significantly higher in the ANA+ group (p = 0.005). The association between ANA status and disease-specific survival did not reach statistical significance (p = 0.054) (**Table 2**).

**Table 2:** ANA positivity and clinical characteristics of COVID-19 patients.

Characteristics	ANA/ENA negative^1^(N = 10)	ANA/ENA positive^2^(N = 12)	p-value
**Age**	**n = 10**	**n = 12**	
years; median (range)	63 (28–88)	70 (53–84)	0.17
**Sex**	**n = 10**	**n = 12**	
Female	7 (70%)	3 (25%)	0.084
Male	3 (30%)	9 (75%)	
**Respiration**	**n = 10**	**n = 12**	
Breathing spontaneously	8 (80%)	1 (8.3%)	**0.002**
Oxygen support	1 (10%)	2 (16.7%)	
Invasive ventilation (IV)	1 (10%)	9 (75%)	
**Duration of IV**	**n = 1**	**n = 9**	
days; median (range)	5	24 (5–39)	na
**Treatment**	**n = 10**	**n = 12**	
peripheral ward	9 (90%)	2 (16.7%)	**0.002**
ICU	1 (10%)	10 (83.3%)	
**Radiological features (CT)^3^**	**n = 6**	**n = 9**	
Typical appearance	3 (50.0%)	5 (55.6%)	1
Indeterminate/Atypical/Negative	3 (50.0%)	4 (44.4%)	
**LDH**	**n = 8**	**n = 12**	
U/L; median (range)	215 (167–348)	374 (248–754)	**0.005**
**CRP**	**n = 8**	**n = 12**	
mg/dl; median (range)	1.1 (0.1–42.1)	11.3 (1–27.6)	0.445
**IL-6**	**n = 9**	**n = 12**	
pg/ml; median (range)	16 (2–2,205)	213 (10–2,093)	0.463
**Outcome**	**n = 10**	**n = 12**	
Follow-up; days; median (range)	69 (7–75)	62 (1–81)	0.376
Dead from disease	1 (10%)	4 (33.3%)	0.323
Severe complications^4^	2 (20%)	9 (75%)	**0.03**

ANA, antinuclear antibody; ARDS, acute respiratory distress syndrome; CRP, C-reactive protein; ICU, intensive care unit; IL-6, interleukin 6; na, not applicable.

^1^ANA/ENA negative: ANA titer <1:320 and negative ENA immunoblot; ^2^ANA/ENA positive: ANA titer ≥1:320 and/or positive ENA immunoblot; ^3^Radiological features have been classified referring to Simpson et al., Radiology: Cardiothoracic Imaging 2.2 (2020): e200152; ^4^Severe complications are summarized in **Supplementary Table 5**.

Significant results (p < 0.05) are highlighted in bold.

### Imaging, Histopathology, and Ultrastructural Analyses

**Figure 3:**
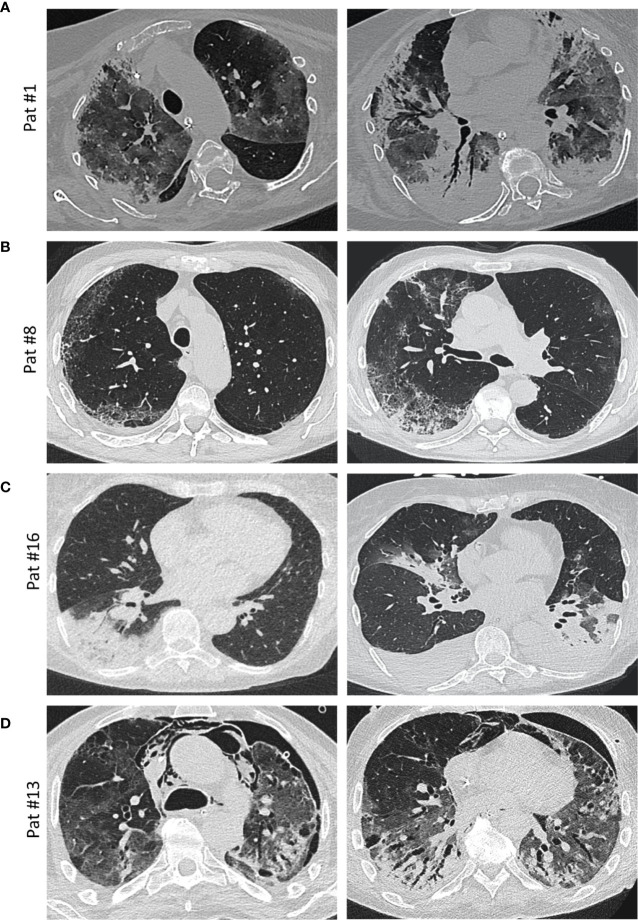
Imaging. **(A)** Axial thin-section unenhanced CT scan of a 69-year-old woman (patient #1, ANA 1:1,000; see **Figure 4A** for histology) 12 days after admission with later-stage disease. Mixed image with diffuse ground-glass opacities (GGOs) and subpleural and peribronchial consolidations with positive aerobrochogram resembling organizing pneumonia. Additionally, anteriorly accentuated irregular subpleural consolidations with partially left-out subpleural space. **(B)** Axial thin-section unenhanced CT scan of a 80 year-old male (patient #8; ANA 1:100), imaged for suspected interstitial lung disease (ILD). Images show right-sided dominant fibrotic changes in the periphery, with partially sparing of the subpleural space, resembling a NSIP-like pattern. Minimal ground-glass-opacities (GGOs) in the left subpleural space. **(C)** Axial thin-section unenhanced CT scans of a 66-year-old female (patient #16, ANA 1:100, anti-Nucleosome/anti-dsDNA positive). Left image was obtained on the day of hospitalization with a typical sign of a lobar pneumonia of the right lower lobe. Right image (day 10 after admission) shows bilateral ground glass opacities and consolidations, mainly on the left lower lobe and the middle lobe. The left lower lobe is properly aerated. Additionally, bilateral pleural effusions are detectable. **(D)** Axial thin-section unenhanced CT scans of a 58-year-old male (patient #13, ANA 1:100, anti-Scl-70 positive; see **Figure 4B** for histology) 4 weeks after onset of the disease and ECMO therapy. In addition to diffuse ground glass opacities, a mixture of bronchiectasis, cysts and air-trapping is evident. Additional pneumothorax and mediastinal emphysema are visible.

**Figure 4:**
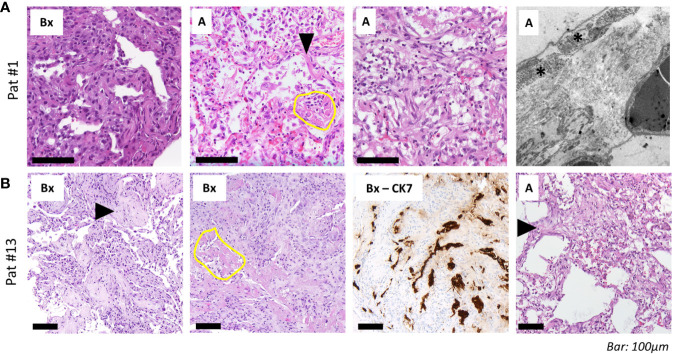
Histopathological and ultrastructural assessment. **(A)** Transbronchial biopsies (“*Bx”*, left) in a 69-year-old woman (patient #1, ANA 1:1,000; see **Figure 3A** for imaging) 13 days after admission shows septal thickening without fibrinous exudate. Tissue samples from the autopsy (“*A”*) of the same patient with reactive pneumocyte changes (“Napoleon hat sign”, arrowhead), ball-like fibrin (yellow circle) and alveoli with plug-like fibromyxoid organization (right). Electron microscopy shows widening of alveolar septa with activated fibroblasts (asterisk) and deposition of collagen (silvery filaments in inter-alveolar septum). **(B)** Transbronchial biopsies in a 58-year-old man (patient #13, ANA 1:100, positive for Scl-70; see **Figure 3D** for imaging) show alveolar fibromyxoid plugs with entrapment of fibrin (“*Bx”*, left; arrowhead). Other areas from the same sample show parenchymal collapse with granulation tissue around residual fibrin (yellow circle). CK7 immunohistochemistry highlights pneumocyte lining of collapsed alveoli. Right: tissue samples from autopsy (“*A*”) show interstitial fibroblast activation (arrowhead) and pseudo-honeycombing. *Scale bar, 100 µm*.

“Typical” radiologic COVID-19 patterns were found in 53.3% of patients (ARDS: 54.4%/non-ARDS: 50%). These included ground glass opacities (all “typical” cases), consolidation and (C)OP-like pattern (**Figure 3**). Atypical/negative patterns were found in 45.6% of ARDS patients and 50% of non-ARDS patients. Bronchoscopy with transbronchial biopsy (TBB) was performed in four patients (cases #1, 8, 13 and 16) before (#8) and after (#1, 13, 16) an established diagnosis of COVID-19, respectively (**Figure 1**). From three of these patients (#1, 8, 13), additional *post-mortem* tissue samples were obtained during a partial autopsy procedure. In all samples, we observed reactive pneumocyte changes (“Napoleon hat sign”) consistent with viral infection (**Figure 4**). However, there was a marked variance in the histologic appearance between different patients, between TBB and autopsy samples from the same patient and between autopsy samples from different regions of the lung. In addition to hyaline membrane formation consistent with diffuse alveolar damage (classic DAD), there was also early septal thickening and intra-alveolar fibrinous plug formation with partial fibromyxoid change, reminiscent of acute fibrinous organizing pneumonia (AFOP) (#1, **Figure 4A**). Ultrastructural analyses of tissue samples from the same patient showed widening of alveolar septa with activated fibroblasts and early deposition of fine collagen fibrils. In patient #13, where biopsies were obtained on day 23 after initial diagnosis, there was a pattern of organizing DAD with parenchymal collapse and entrapment of fibrin (**Figure 4B**). Tissue samples from autopsy from the same patient showed areas of beginning, patchy fibrosis with a foreshadowing of honeycombing. In all autopsy samples, there was capillary congestion with formation of microthrombi especially in late-stage disease (**Supplementary Figure 1**).

## Discussion

In the present study, we found overlapping serological, clinical, radiologic, and histopathological features of severe COVID-19 and lung manifestation of autoimmune disease (CTD-ILD). We show that presence of ANAs is significantly associated with the development of ARDS, necessity for ICU treatment and invasive ventilation as well as occurrence of severe complications in these patients; noteworthy, every patient in the present study who presented with or developed ARDS had detectable autoantibodies. With respect to baseline clinical characteristics, the investigated cohort is comparable to previous reports (2, 19). Our result of autoantibodies in patients with severe COVD-19 is in line with first results from other groups (10, 11, 20); however, we are the first to put this observation into context with clinical, imaging, and histopathology findings. Furthermore, we confirm the association between higher age, male sex, and elevated LDH with severe course of COVID-19 in line with literature data (19). Given the fact that only hospitalized patients were included, it is not surprising that the mortality rate (22.7%) was higher compared to the general population.

Imaging and histopathological data in the present and in previous studies show that presentation of COVID-19 in the lung is heterogeneous and evolves over time (4, 21–23). Overall diversity of these changes, including (organizing) diffuse alveolar damage, fibromyxoid plugging and interstitial thickening are reminiscent of exacerbation of CTD (24, 25). However, it has to be clearly acknowledged that the histopathological spectrum of virus-induced DAD is wide and also includes findings that have recently been described to be specific for COVID-19, such as endothelialitis and (micro-)thrombotic events. Our finding that significant ANA titers and/or detection of specific autoantibodies are found in most patients who develop ARDS raises the question if there is a comparable mechanism of lung damage between SARS-CoV-2 infection and exacerbating autoimmune disease. In 4/6 COVID-19 patients with specific ENAs who developed ARDS, detected autoantibodies were anti-PM-Scl or anti-Scl-70; if the borderline positivity for RP155 in patient #9 was included, 5/6 specific ENAs in our cohort would be associated with a form of sclerosing CTD, as these autoantibodies (as well as similar HR-CT) patterns have previously been described in dermatomyositis, (progressive) systemic sclerosis and CTD-overlap syndromes (26, 27). Of note, a significant proportion of anti-PM-Scl-/anti-Scl-70-positive patients develop pulmonary fibrosis, raising the question of long-term effects of severe COVID-19 in these patients (28). The possibility of progressively evolving fibrosis would be supported by our findings from histopathology and electron microscopy, where we observed organization and pseudo-honeycombing as well as interstitial fibroblast activation with collagen deposition. Another parallel between CTDs and COVID-19 are the vasculitis-like changes, vascular dysfunction or microangiopathies that have been described in a subset of patients (29–31). While thromboembolic complications occurred in only two patients in our cohort (both ANA-positive), it would be of great interest to screen patients with more widespread vascular or cutaneous involvement for the presence of ANA. ANCA screening, however, was completely negative in our cohort of COVID-19 patients.

Since it is well-known that ANA screening can be false positive in severely ill patients or patients who undergo ICU treatment, a possible epiphenomenon has to be discussed very frankly. According to a recent publication, a there is no cross-reactivity between anti-SARS-CoV-2 IgG/IgM and CTD-associated autoantibodies (32). However, to enhance test validity in the present study, we a) doubled the recommended threshold for ANA screening (9) from 1:160 to 1:320 and b) performed additional immunoblot for specific ENA in all patients, thus adding an independent methodological approach combining high sensitivity and specificity (16, 33). Moreover, we included a non-COVID-19 pneumonia control group in which ANA titers ≥ 1:320 could be detected in two patients. Specific autoantibodies against AMA-M2 (associated with primary biliary cirrhosis) and Ro52 (associated with SLE) were detected in two additional patients. Laboratory values in the pneumonia control group (LDH, CRP, IL-6) were not significantly different compared to COVID-19 ARDS cases. While it is conceivable that ANA titers rise and specific autoantibodies may appear in severely ill patients in general, we think that the observed clustering of high ANA titers with specific, sclerosis-associated autoantibodies in the COVID-19 ARDS group is reliable, raising the question of how these autoantibodies arise in the context of SARS-CoV-2 infection.

A recent preprint suggests significant extrafollicular B cell activation with an excessive production of antibody-secreting cells (ASCs) in critically ill SARS-CoV-2 patients (34). This mechanism is highly similar to the development and progression of SLE, and these ASC might represent a possible source of the autoantibodies we report here (35, 36). We do not assume that these autoantibodies were already present in predisposed patients prior to infection, because only two patients in our cohort had any clinical history of rheumatic or autoimmune disease. However, in light of our results, there are interesting parallels between the reported epidemiology of severe COVID-19 and the presence of autoantibodies in the general population. Autoantibody titers above 1:80 and 1:160 can be detected in 13.3 and 5% of otherwise healthy individuals (37), reflecting reported proportions of severe (14%) and critical (5%) course of COVID-19 (38). Preliminary reports from the U.S. suggest that the COVID-19-associated death rate among African Americans is significantly higher compared to the general population (39), while at the same time ANA titers in African Americans exceed those of Americans with another ethnic background (40). It would be interesting to screen patients for class I and class II major histocompatibility complex antigens to see whether it is possible to identify patients with an enhanced risk for development of autoantibodies and severe course of the disease.

There is an ongoing debate with regard to a possible dysregulation of the immune system by SARS-CoV-2, and it has been discussed whether anti-inflammatory drugs might be beneficial to prevent potentially harmful hyperinflammation (41). Our hypothesis of SARS-CoV-2-induced immune dysregulation closely correlates with results from the Wuhan cohort reported by Wu et al., in which methylprednisolone treatment was associated with a more favorable outcome among the patients who had already developed ARDS (19). A recent report from Japan described high anti-SSA/Ro antibody titers in two patients with severe COVID-19 pneumonia, one of which responded well to corticosteroid therapy (42). Moreover, first results from the UK RECOVERY trial (EudraCT 2020-001113-21, press release from Oxford University on June 16, 2020) indicate a significant benefit for dexamethasone, a drug that is also in use for the treatment of SSc, in mechanically ventilated patients. It would furthermore be interesting to evaluate if patients with SLE-like ANA pattern (anti-ds-DNA) profit from hydroxychloroquine, while patients with an SSc-like ANA pattern (anti-Scl-70, anti-CENP) might respond to cyclophosphamide. In line with that, one case report described a mild clinical course of COVID-19 in patient with established anti-Scl-70-positive SSc under treatment with the anti-interleukin (IL) 6 receptor blocker tocilizumab (43). The correct timing and dosing for any immunosuppressive or anti-fibrotic treatment approach in response to a viral infection however remains unclear.

Possible limitations of this study include its limited sample size and the lack of randomization. A further limitation of this study is the possibility of selection bias, which could not be ruled out on account of the study design.

Our observation of CTD-associated autoantibodies together with the CTD-like radiologic and histopathologic lung findings in severe cases of COVID-19 point towards a possible dysregulation of the immune response upon SARS-CoV-2 infection that might fuel organizing pneumonia and trigger interstitial fibrosis, with deleterious effects on the functional outcome in long-term survivors. Early detection of the reported autoantibodies might identify patients who profit from immunosuppressive and/or anti-fibrotic therapy to prevent the development of respiratory failure and fibrosis in COVID-19.

## Data Availability Statement

All datasets presented in this study are included in the article/**Supplementary Material**.

## Ethics Statement

The studies involving human participants were reviewed and approved by the Ethics committee of the University of Ulm (ref. no. 129-20). The patients/participants or their relatives provided their written informed consent to participate in this study. Written informed consent was obtained from the individual(s) for the publication of any potentially identifiable images or data included in this article.

## Author Contributions

Study concept: DG and KS. Data collection: HW, DG, BS, MH, CR, WB, VU, JS, and KS. Sample collection: DG, VU. Statistical analysis: HW, NG, and KS. Initial draft of manuscript: KS, JS, DG, and HW. All authors contributed to the article and approved the submitted version.

## Conflict of Interest

KS serves on advisory boards for MSD, Novartis and Bristol-Myers Squibb (BMS). KS, CH, and DG were speakers for Boehringer-Ingelheim. KS has received travel reimbursements from PharmaMar. DG serves on advisory boards for Novartis, Boehringer Ingelheim, Berlin Chemie, MSD, Roche and AstraZeneca. JS reports personal fees from Novartis, Astellas, BMS, MSD, Roche, Bayer, Ipsen and Janssen, outside the submitted work.

The remaining authors declare that the research was conducted in the absence of any commercial or financial relationships that could be construed as a potential conflict of interest.
