# Supplementary figures and images for: Clinical, Serological, and Histopathological Similarities Between Severe COVID-19 and Acute Exacerbation of Connective Tissue Disease-Associated Interstitial Lung Disease (CTD-ILD)
Source: Front Immunol. 2020 Oct 2;11:587517. doi: 10.3389/fimmu.2020.587517 (PMC7566417; doi:10.3389/fimmu.2020.587517)

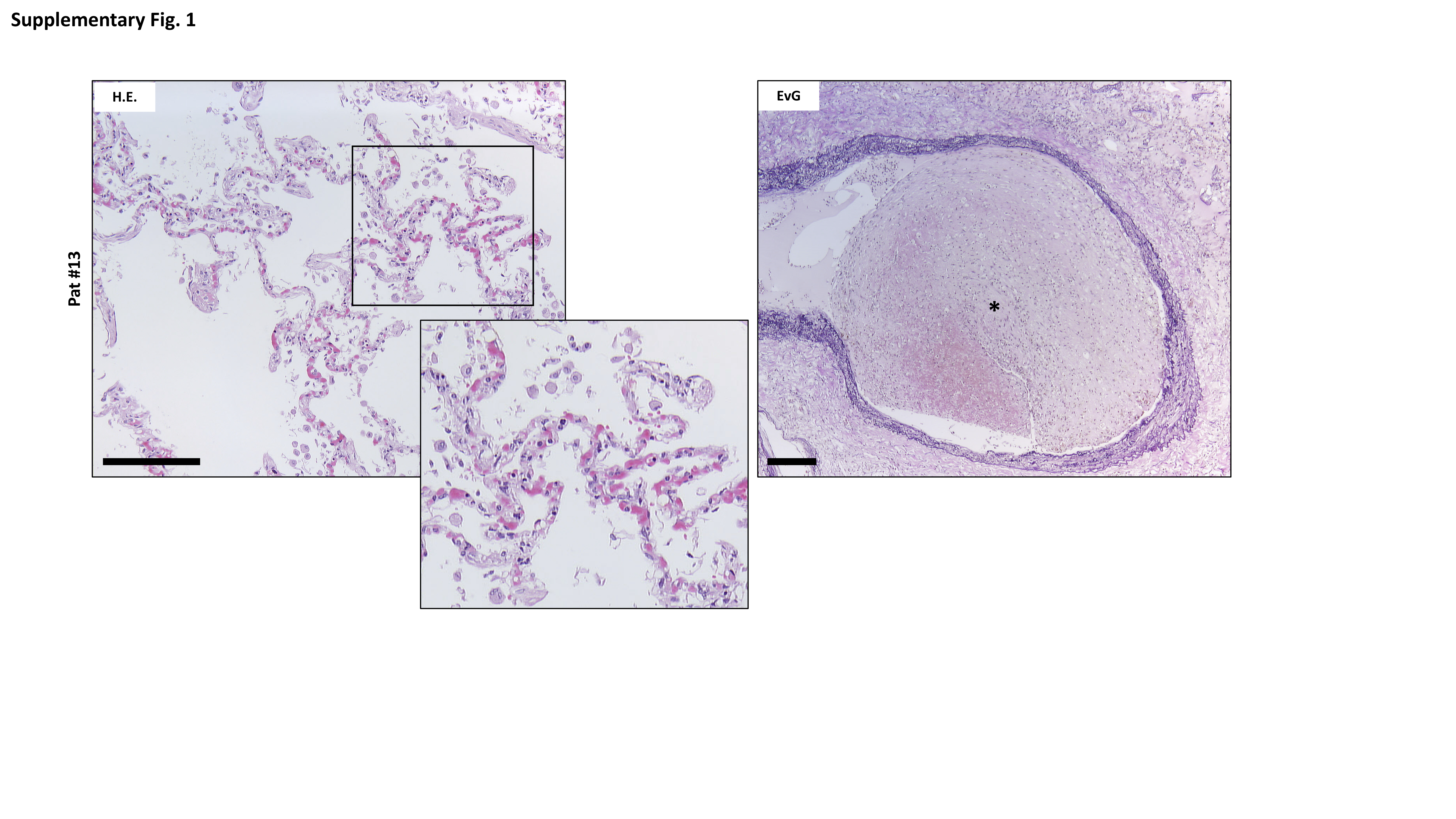

Supplement: Supplementary file 1 [file Image_1.jpeg]
