# Supplementary material for: Clinical, Serological, and Histopathological Similarities Between Severe COVID-19 and Acute Exacerbation of Connective Tissue Disease-Associated Interstitial Lung Disease (CTD-ILD)
Source: Front Immunol. 2020 Oct 2;11:587517. doi: 10.3389/fimmu.2020.587517 (PMC7566417; doi:10.3389/fimmu.2020.587517)
Supplement: Supplementary file 2 [file Table_1.docx]

***Supplementary Material***

**Supplementary Table 1.** Clinical characteristics of the non-COVID-19-pneumonia control group

| **Characteristics** | **All patients**  **N=10** |
| --- | --- |
| **Age** | **n=10** |
| years; median (range) | 76 (35-91) |
| **Sex** |  |
| Female | 6 (60%) |
| Male | 4 (40%) |
| **Preexisting diseases** | **n=10** |
| 0 | 2 (20%) |
| ≥1 | 8 (80%) |
| **Type of preexisting disease** | **n=10** |
| Cardiovascular risk factors^1^ | 8 (80%) |
| Cardiovascular disease^2^ | 5 (50%) |
| Oncological disease | 4 (40%) |
| Rheumatic disease | 0 |
| **Imaging at initial diagnosis** | **n=10** |
| CT scan | 10 (100%) |
| **ICU treatment** | **n=10** |
| Yes | 1 (10%) |
| No | 9 (90%) |
| **Respiration** | **n=10** |
| Breathing spontaneously | 4 (40%) |
| Oxygen support | 5 (50%) |
| Invasive ventilation | 1 (10%) |
| **LDH** | **n=10** |
| U/l; median (range) | 215 (115-558) |
| **C-reactive protein** | **n=10** |
| mg/dl; median (range) | 8.6 (0.1-20.0) |
| **IL-6** | **n=10** |
| pg/ml; median (range) | 118 (14-42586) |
| **ANA/ENA (IIF+IB)** | **n=10** |
| ANA/ENA neg.^3^ | 6 (60%) |
| ANA/ENA pos.^4^ | 4 (40%) |
| **Outcome** | **n=10** |
| Follow-up: days; median (range) | 12 (1-37) |
| Dead from disease | 2 (20%) |
| Severe complications^6^ | 3 (30%) |
| ANA, antinuclear autoantibody; CT, computed tomography; ENA, extractable nuclear antigen; IB, immunoblot; IIF, indirect immunofluorescence; SARS-CoV-2, severe acute respiratory syndrome coronavirus 2. ^1^diabetes mellitus, dyslipidemia, arterial hypertension, obesity, nicotine abuse; ^2^coronary disease, post-myocardial infarction, peripheral arterial vaso-occlusive disease, post-stroke, atherosclerosis; ^3^ANA/ENA negative: ANA titer <1:320 and negative ENA immunoblot; ^4^ANA/ENA positive: ANA titer ≥1:320 and/or positive ENA immunoblot; ^5^acute renal failure, septic cardiomyopathy, lactic acidosis. | |

**Supplementary Table 2.** Antiviral treatment approaches.

| **ID** | **Age band** | **Sex** | **Treatment approach** | **Duration of treatment** | **Toxicity** |
| --- | --- | --- | --- | --- | --- |
| 1 | 60s | f | HCQ + Azithromycin | 10 days | None |
| 3 | 50s | m | Kaletra® | 2 days | Acute kidney injury |
| 3 | 50s | m | HCQ + Azithromycin | 2 days | Drug related exanthema |
| 6 | 60s | m | HCQ + Spiramycin | 4 days | Acute kidney injury |
| 7 | 70s | m | Kaletra® | 2 days | None |
| 7 | 70s | m | HCQ + Spiramycin | 6 days | None |
| f, female; HCQ, hydroxychloroquine; m, male. | | | | | |

**Supplementary Table 3.** Intensive care treatment of COVID-19 patients.

| **Characteristics** | **COVID-19 ICU patients (n = 11)** |
| --- | --- |
| **Duration of ICU treatment** | |
| Days (median + range) | 24 (7 - 39) |
| **Organ failure (apart from ARDS)** | |
| Renal failure | 4 (36.4%) |
| Dialysis required | 1 (9.1%) |
| Hepatic failure | 4 (36.4%) |
| Cardiac failure | 2 (18.2%) |
| Septic shock | 4 (36.4%) |
| **Ventilation** | |
| Invasive ventilation (IV) | 10 (90.9%) |
| TTI (days; median + range) | 2.5 (1 - 6) |
| Duration of IV (days; median + range) | 23.5 (5 - 39) |
| Highest PEEP (mbar; median + range) | 15 (6 - 20) |
| Highest Pinsp (mbar; median + range) | 29 (18 - 34) |
| Worst FiO_2_ (%; median + range) | 55 (30 - 80) |
| Lowest compliance (ml/cm H_2_O; median + range) | 45.5 (30 - 70) |
| Prone positioning | 7 (63.6%) |
| Prone positioning success rate | 5/7 (71.4%) |
| Extubation success rate | 2/10 (20.0%) |
| Tracheostomy rate | 6/10 (60.0%) |
| Murray lung injury score (median + range)* | 2.88 (2.00 - 3.50) |
| **Circulation** | |
| Catecholamine support (CAS) | 10 (90.9%) |
| Duration of CAS (days; median + range) | 12.5 (2 - 30) |
| **Outcome** | |
| ICU-mortality | 4 (36.4%) |
| Discharged from ICU | 7 (63.6%) |
| ARDS, acute respiratory distress syndrome; ICU, intensive care unit; CAS, catecholamine support; PEEP, positive end-expiratory pressure; Pinsp, inspiratory pressure; TTI, time-to-intubation  *Murray et al., Am Rev Respir Dis 138.3 (1988): 720-723. | |

**Supplementary Table 4.** Laboratory findings and cut-off values in COVID-19 patients.

| **Laboratory characteristics** | **Cut-off value/ units** | **Results; median (range)** |
| --- | --- | --- |
| Lymphocytes | Per nanoliter | d0 = 720 (320 - 2540) |
|  |  | d5 = 830 (610 - 3210) |
|  |  | d10 = 900 (500 - 4300) |
|  |  | d15 = 1270 (620 - 8320) |
| Neutrophil granulocytes | Per nanoliter | 4520 (1080 - 12300) |
| Lactate dehydrogenase | ≥ 240.0 U/l | 297.5 (167 - 754) |
| D-dimers | ≥ 0.50 mg/l | 1.48 (0.31 - 30.00) |
| Fibrinogen | ≥ 0.50 g/l | 5.16 (2.74 - 9.00) |
| Bilirubin | ≥ 0.50 mg/dl | 0.69 (0.23 - 5.41) |
| Albumin | ≤ 35.0 g/l | 29.5 (18.0 - 49.0) |
| C-reactive protein | ≥ 0.50 mg/dl | 7.25 (0.10 - 42.10) |
| Procalcitonin | ≥ 0.50 ng/ml | 0.35 (0.10 - 5.30) |
| Interleukin-6 | ≥ 10 pg/ml | 61 (<2 - 2205) |
| Creatinine | ≥ 0.90 mg/dl | 0.98 (0.31 - 5.41) |
| Troponin T | > 0.013 ng/ml | 0.015 (0.013 - 0.464) |
| Ferritin | ≥ 400.0 ng/ml | 845.5 (40.0 - 3483.0) |

**Supplementary Table 5.** Antibiotic treatment modalities due to suspected superinfection in COVID-19 patients.

| **ID** | **Age band** | **Sex** | **Antibiotic agent** | **Pathogen detection** | **Focus** |
| --- | --- | --- | --- | --- | --- |
| 1 | 60s | f | Ceftriaxone | None | Respiratory tract |
| 3 | 50s | m | Meropenem | None | Respiratory tract |
| 5 | 50s | m | Piperacillin/tazobactam | None | Respiratory tract |
| 6 | 60s | m | Meropenem | None | Respiratory tract |
| 8 | 80s | m | Piperacillin/tazobactam | None | Respiratory tract |
| 9 | 70s | m | Piperacillin/tazobactam | None | Respiratory tract |
| 13 | 50s | m | Piperacillin/tazobactam  Vancomycin | Staphylococcus aureus | Sepsis,  respiratory tract |
| 13 | 50s | m | Meropenem | Klebsiella pneumonia | Sepsis  respiratory tract, urogenital tract |
| 14 | 80s | m | Piperacillin/tazobactam | E. coli | Respiratory tract, urogenital tract |
| 16 | 60s | f | Meropenem | None | Respiratory tract |
| 18 | 80s | m | Ampicillin/sulbactam | None | Respiratory tract |
| 19 | 80s | m | Ampicillin/sulbactam | None | Respiratory tract |

**Supplementary Table 6**. Therapy- or disease-related complications in COVID-19 patients.

| **ID** | **Age band** | **Sex** | **Day** | **Complication** | **Cause** |
| --- | --- | --- | --- | --- | --- |
| 1 | 60s | f | 12 | Acute kidney injury | Disease-related |
| 3 | 50s | m | 9 | Acute kidney injury | Therapy-related |
| 3 | 50s | m | 17 | Rectal arterial bleeding, thrombosis | Disease-related |
| 5 | 50s | m | 7 | Ventilator associated pneumonia | Therapy-related |
| 5 | 50s | m | 7 | Septic shock | Therapy-related |
| 6 | 60s | m | 5 | Acute kidney injury | Therapy-related |
| 6 | 60s | m | 5 | Septic shock | Disease-related |
| 6 | 60s | m | 10 | Dialysis | D + T-related |
| 9 | 70s | m | 2 | Congestive hepatitis | Disease-related |
| 13 | 50s | m | 9 | Veno-venous ECMO* | Disease-related |
| 13 | 50s | m | 18 | Pneumothoax | Therapy-related |
| 13 | 50s | m | 29 | Ventilator associated pneumonia | Therapy-related |
| 14 | 80s | m | 1 | Acute on chronic kidney injury | Disease-related |
| 14 | 80s | m | 1 | Cardiac decompensation | Disease-related |
| 15 | 20s | f | 18 | Fatigue | Disease-related |
| 16 | 60s | f | 3 | Septic shock | Disease-related |
| 16 | 60s | f | 10 | Pneumothorax | Therapy-related |
| 18 | 80s | m | 1 | Pulmonary embolism | Disease-related |
| D, disease; ECMO, extracorporeal membrane oxygenation; f, female; m, male; T, therapy. * Patient transferred to another institution for vv-ECMO treatment approach. | | | | | |

**SUPPLEMENTARY FIGURE LEGENDS**

**Sup Fig. 1.** In all autopsy samples, there was capillary congestion with formation of microthrombi especially in late-stage disease.

**List of Abbreviations**

| **Abbreviation** | **Explanation** |
| --- | --- |
| AAB | autoantibody |
| AFOP | acute fibrinous organizing pneumonia |
| AMA | anti-mitochondrial antibodies |
| ANCA | anti-neutrophil cytoplasmatic antibodies |
| ANA | antinuclear antibody |
| APC | antigen-presenting cells |
| ARDS | acute respiratory distress syndrome |
| BAL | bronchoalveolar lavage |
| CD | cluster of differentiation |
| CENP-B | centromere protein B |
| CMV | cytomegalovirus |
| COVID-19 | coronavirus disease 2019 |
| CRP | C-reactive protein |
| CT | computed tomography |
| CTD-ILD | connective tissue disease |
| CTL | cytotoxic T-lymphocytes |
| DAD | diffuse alveolar damage |
| DNA | deoxyribonucleic acid |
| EBV | Epstein Barr virus |
| ENA | extractable nuclear antibodies |
| EvG | Elastica-van-Gieson |
| HE | haematoxylin-eosin |
| HR-CT | high-resolution computed tomography |
| IB | immunoblot |
| ICU | intensive care unit |
| IFNα | interferon α |
| IIF | indirect immunofluorescence |
| IL-6 | interleukin-6 |
| LDH | lactate dehydrogenase |
| MG | Masson-Goldner |
| MPO | myeloperoxidase |
| OP | organizing pneumonia |
| PBS | phosphate buffered saline |
| PCNA | proliferating cell nuclear antigen |
| PCT | procalcitonin |
| PM-Scl | polymyositis-scleroderma |
| PR3 | proteinase 3 |
| PSS | progressive systemic sclerosis |
| PTAH | phosphotungstic-acid-hematoxylin |
| RNA | ribonucleic acid |
| RNP | ribonucleoprotein particles |
| RSNA | radiological Society of North America |
| RT-PCR | real-time polymerase chain reaction |
| SARS-CoV-2 | severe acute respiratory syndrome coronavirus 2 |
| SLE | systemic lupus erythematosus |
| TBB | transbronchial biopsy |
| TLR | toll-like receptors |
| vv-ECMO | veno-venous extracorporeal membrane oxygenation |
